# Impact of depth-of-interaction and spatially-variant point-spread-function model on the performance of ultra-high resolution brain PET

**DOI:** 10.1088/1361-6560/aeafa5

**Published:** 2026-10-16

**Authors:** Tianyi Zeng, Yassir Najmaoui, Jiazhen Zhang, Tommaso Volpi, Kathryn Fontaine, Yanis Chemli, Yifan Hu, Jean-Dominique Gallezot, Chen Sun, Yihuan Lu, Georges El Fakhri, Richard E Carson, Thibault Marin

**Affiliations:** 1 Department of Radiology and Biomedical Imaging, Yale School of Medicine, New Haven, CT, United States of America; 2 Yale Biomedical Imaging Institute, Yale School of Medicine, New Haven, CT, United States of America; 3 Department of Biomedical Engineering, Yale University, New Haven, CT, United States of America; 4 United Imaging Healthcare, Shanghai, People’s Republic of China

**Keywords:** positron emission tomography (PET), spatially-variant point spread function (PSF), depth-of-interaction (DOI), open-source reconstruction platform

## Abstract

*Objective.* To implement a spatially-variant point spread function (VarPSF) model for the NeuroEXPLORER (NX) brain positron emission tomography (PET) scanner in the open-source Yale Reconstruction Toolkit for PET, and investigate the interplay between depth-of-interaction (DOI) resolution and resolution modeling. *Approach.* PSF parameters were estimated from simulated and physically measured point sources. Three primary DOI configurations were evaluated: eight-layer DOI (8DOI), two-layer DOI (2DOI), and no DOI (noDOI), with an additional 4DOI comparison in the off-center simulated Derenzo study. Reconstructions using VarPSF, a spatially-invariant PSF measured at the field-of-view center (SinglePSF), and no PSF modeling (noPSF) were assessed using simulated Derenzo and brain phantoms, a physical Derenzo phantom, and one non-human primate (NHP) scan. *Main results.* In simulated and physical Derenzo studies, VarPSF yielded higher peak-to-valley ratios than SinglePSF and noPSF at the final evaluated iterations, with pronounced gains at large radial offsets. In the simulated brain phantom, VarPSF reduced the estimated transverse blur by 0.3 mm relative to SinglePSF and 0.5 mm relative to noPSF in 2DOI, and by 0.6 mm and 0.8 mm, respectively, in noDOI. In the NHP study, VarPSF improved contrast and structural delineation with reduced DOI encoding at approximately matched white-matter coefficient of variation, while its incremental benefit over SinglePSF was small with 8DOI at clinically relevant iteration settings. *Significance.* Spatially-variant PSF modeling can partially compensate for limited DOI information. The findings motivate combining modest DOI capability with accurate resolution modeling, while further validation is required for redesigned detectors and fully corrected acquisitions. The open-source implementation enables controlled investigation of the complementary roles of DOI encoding and resolution modeling.

## 1. Introduction

Positron emission tomography (PET) plays an important role in both clinical studies and neuroscience research. As a powerful molecular imaging tool, brain PET is critical for diagnosing and monitoring a range of neurological and psychiatric disorders, including Alzheimer’s disease, Parkinson’s disease, and epilepsy (Johnson *et al* 2013, Matuskey *et al* 2020, Xiao *et al* 2024). In neuroscience research, brain PET enables quantitative assessment of cerebral blood flow, metabolism, and receptor occupancy, and serves as a key tool for investigating brain pharmacology and physiology (Mosconi 2013, Okazawa *et al* 2018, Khodaii *et al* 2022, Carson *et al* 2023).

As our understanding of finer brain structures and functional subregions continues to advance, brain PET development has increasingly focused on dedicated systems that can deliver both high spatial resolution and high sensitivity (Jones and Rabiner 2012, Catana 2019). The well-known high resolution research tomograph (HRRT), designed to achieve a spatial resolution of at least 2.5 mm across the human brain, has played a central role in advancing high-resolution PET imaging for neuroscience and clinical research, with 17 systems installed worldwide (Wienhard *et al* 2002, Schain *et al* 2013). More recently, multiple high-performance dedicated brain PET systems have been developed, further enhancing image resolution, sensitivity, and quantitative performance. These systems have further strengthened the role of quantitative PET in neurology, including early diagnosis of neurodegenerative disorders and detailed studies in neuropsychiatry and neurooncology (Onishi *et al* 2022, Zeng *et al* 2023, Fang *et al* 2024, Li *et al* 2024, Doyon *et al* 2025). Among these advances, we developed a next-generation ultra-high-performance brain PET scanner, NeuroEXPLORER (NX). NX features a long axial field-of-view (FOV) (49.5 cm) and high-resolution detectors (1.64 mm radial and 2.73 mm axial spatial resolution) with eight-layer depth-of-interaction (DOI) encoding based on a single-ended detector readout, enabling both high sensitivity and high spatial resolution across the full FOV (Li *et al* 2024). Compared with HRRT in the same subjects across seven tracers, NX showed substantially improved image detail, particularly in small brain structures (Volpi *et al* 2026). In parallel, alternative geometries, such as helmet PET and dedicated PET/MR-compatible inserts, have also been proposed and clinically evaluated, offering improved sensitivity and a promising path toward high-resolution, cost-effective brain imaging (Tashima and Yamaya 2016, Won *et al* 2021, Akamatsu *et al* 2022, Dong *et al* 2024, Kuang *et al* 2024).

To maintain uniform spatial resolution across the PET FOV, DOI techniques are commonly used. DOI is a detector technology that estimates the depth at which a gamma photon interacts within the scintillation crystal, thereby reducing parallax error and preserving high and spatially uniform resolution across the full imaging field (Peng and Levin 2010). This capability is particularly important for dedicated brain PET, where small structures and subtle regional differences place stringent demands on image resolution (Carson and Kuo 2019). Several DOI detector architectures have been developed for high-resolution PET, including multilayer, dual-ended readout, and single-ended detectors. Multilayer approaches encode interaction depth by dividing the detector crystal depth into discrete scintillator layers and have been implemented in brain PET systems using two- or four-layer detector configurations (Wienhard *et al* 2002, Watanabe *et al* 2017, Nishikido *et al* 2022). Dual-ended approaches estimate DOI from the relative light collected at opposite ends of the crystal (Kuang *et al* 2024), whereas single-ended approaches infer interaction depth from depth-dependent light-sharing or light-distribution characteristics measured from one side of the detector (Lee and Lee 2015, Kuang *et al* 2018, Zeng *et al* 2023). NX specifically adopts a U-shaped axial light-sharing crystal architecture coupled to a single-ended SiPM array, providing eight DOI bins over the 20-mm crystal depth (Li *et al* 2024). Each detector microblock consists of four U-shaped elements coupled to a 2×2 SiPM array, and DOI is estimated from the relative scintillation-light distribution measured by the two axial SiPMs. This NX-specific implementation combines eight-bin DOI estimation with single-sided photodetector readout in a compact high-resolution detector geometry.

Accurate quantitative PET imaging requires explicit modeling of the physical factors that determine image resolution (Ashrafinia *et al* 2017). In brain PET, spatial resolution is fundamentally limited by detector and interaction physics, including crystal size, crystal cross-section, DOI uncertainty, positron range, and photon noncollinearity. Resolution modeling compensates for the resulting blur during image reconstruction. A widely used framework is point spread function (PSF) modeling, in which the PSF describes how the system distorts an ideal point source (Rahmim *et al* 2013). In practice, PSF can be estimated and incorporated in the projection space (Tohme and Qi 2009), image space (Zhou and Qi 2014) or both (Deller *et al* 2021). Importantly, PET spatial resolution is not uniform across the FOV. Due to effects such as oblique photon incidence at larger radial offsets, the PSF is spatially-variant and generally broadens toward the edge of the FOV (Zhang *et al* 2016). Accordingly, multiple methods have been proposed to measure and implement spatially-variant PSF models (Kotasidis *et al* 2010, Rapisarda *et al* 2010, Gravel *et al* 2019). For iterative-reconstruction-based PSF measurement, point sources with warm background are recommended, since measurements without background tend to underestimate the PSF parameters, resulting in smaller estimated PSF full width at half maximum (FWHM) values (De Jong *et al* 2007, Gong *et al* 2016). Recent simulation studies further demonstrate that DOI resolution and spatially-variant PSF modeling are both critical for achieving high-resolution, high-sensitivity brain PET performance (Gao *et al* 2026). Therefore, spatially-variant PSF modeling is essential for accurately capturing location-dependent system response and for improving resolution uniformity and quantitative fidelity across the full FOV.

Although spatially-variant resolution modeling is included in the vendor NX reconstruction and has been used in prior NX studies (Li *et al* 2024, Hu *et al* 2026, Volpi *et al* 2026), its implementation is system-specific and does not readily enable controlled evaluation of the respective contributions of DOI resolution and PSF modeling. To address this need, this work implements a spatially-variant PSF model for NX in the open-source Yale Reconstruction Toolkit for Positron Emission Tomography (YRT-PET) (Najmaoui *et al* 2026). This implementation provides a transparent and reproducible framework for controlled evaluation of the interaction between DOI resolution and spatially-variant PSF modeling on NX.

Specifically, we first use a simulation-based method (Gong *et al* 2016) to estimate the system resolution response from point sources with background and develop a spatially-variant PSF model for three primary DOI configurations. The reconstructed point sources are fitted with 3D Gaussian models to obtain PSF parameters, which are stored in lookup tables (LUTs); during reconstruction, local PSF parameters are selected from the LUT using nearest-neighbor interpolation. Second, we quantitatively evaluate how different DOI resolutions and PSF modeling strategies affect image quality using Monte Carlo simulation (MCS) studies, including a targeted comparison with an intermediate 4DOI configuration under an off-center condition. Third, we validate these findings using a physical mini-Derenzo phantom and *in-vivo* non-human primate (NHP) NX scans by assessing the practical impact of different DOI and PSF configurations under realistic imaging conditions.

## 2. Methods

### 2.1. Reconstruction and simulation platforms

In this study, two platforms were used for NX data simulation and reconstruction: the United-Imaging molecular imaging innovation platform (uMIP) (Sun *et al* 2023) and the YRT-PET (Najmaoui *et al* 2026). uMIP provides a GPU-based MCS toolkit reported to be up to three orders of magnitude faster than CPU-based GATE implementations, supports NX-specific scanner geometry with customizable source and system parameters, and generates list-mode PET data while modeling key physical effects such as positron range, photon noncollinearity, scatter, and detector interactions. For image reconstruction, YRT-PET is an open-source C++ reconstruction engine with CUDA acceleration and Python bindings. It is designed to support multiple scanners and data formats via its plugin architecture that separates scanner-specific I/O from the core reconstruction pipeline (Najmaoui *et al* 2025a). YRT-PET implements corrections for degrading factors in PET including normalization, attenuation, and event-by-event motion correction. The spatially-variant PSF model described in this work was implemented in YRT-PET.

### 2.2. DOI configuration settings

To evaluate the impact of DOI encoding on reconstruction performance, we implemented three primary DOI configurations in YRT-PET, all based on the same NX scanner geometry. For all three configurations, the crystal depth was fixed and the underlying NX detector simulation was kept unchanged, while the DOI information provided to the reconstruction model was modified. The first configuration was the 8DOI setting. For the original 8DOI MCS, photon interaction positions and deposited energies within the detector crystals were simulated. For each micro-block, an energy-weighted average interaction position was calculated to determine the theoretical DOI. A Gaussian-distributed DOI uncertainty with a FWHM of 3.2 mm, based on the measured system-average DOI response, was then applied to the theoretical DOI position. The DOI estimation was then discretized into eight DOI bins with a width of 2.5 mm. Thus, the 2.5 mm value represents the DOI binning interval and not the intrinsic DOI resolution. The physical NX system similarly reports eight DOI indices over the 20 mm crystal depth with finite DOI uncertainty (Li *et al* 2024). The second configuration was a simplified two-layer model (2DOI), constructed from the layer-count distribution of the 8DOI setting, with front- and back-layer thicknesses of 7.5 mm and 12.5 mm selected to obtain a balanced 50%/50% count split between the two layers. The third configuration was a no-DOI baseline (noDOI), in which a single layer was used and no depth information was provided. The list-mode data were first generated in the 8DOI configuration. To form the 2DOI and noDOI datasets, each line-of-response (LOR) endpoint was reassigned from its original 8DOI bin to the center of the closest layer of the new configuration, and its DOI index was updated accordingly.

Accordingly, the simulated 2DOI and noDOI datasets retained the photon-interaction histories and detector-response characteristics of the original 8DOI MCS; only the DOI information supplied to reconstruction was coarsened, and normalization factors were recalculated for each DOI definition. These datasets should therefore be interpreted as virtual reconstruction-level DOI conditions rather than end-to-end simulations of physically redesigned 2DOI or noDOI detectors. The 2DOI setting was selected to represent coarse DOI encoding with practical precedent in high-resolution brain PET, including the two-layer HRRT detector (De Jong *et al* 2007). An additional 4DOI configuration was included for a targeted off-center Derenzo comparison, as described in section 3.1.

### 2.3. Spatially-variant PSF measurement and implementation

For simulation studies, spatially-variant PSF parameters were measured using a uMIP MCS point-source (80 kBq activity) array with 25 mm spacing over a 200 mm range along all three axes. A uniform background was added, with the background activity concentration set to 90% of the point-source, following the procedure in Gong *et al* (2016). For each DOI configuration (8DOI, 4DOI, 2DOI, and noDOI), the simulated data were reconstructed using 50 maximum-likelihood expectation-maximization (MLEM) (Lange and Carson 1984) iterations without PSF modeling to avoid biasing the measured system response. An anti-aliasing filter was applied to mitigate discretization artifacts. Point-source-only images were then obtained by subtracting a background-only reconstruction from the corresponding point-source-with-background reconstruction. Using an isotropic voxel size of 0.6 mm, each isolated point source was fitted with a 3D Gaussian model, and the resulting FWHM values along the three spatial directions were extracted as PSF parameters.

For real NX *in-vivo* studies, spatially-variant PSF parameters were measured using a Na-22 point source (80 kBq activity) at different locations, with 50 mm spacing over a 200 mm range along each axis independently. The same PSF measurement method was used as in simulation, except that no background was included. Based on the results shown in figure 1, the 10th MLEM iteration was empirically selected for real NX PSF fitting to reduce the risk of iteration-dependent PSF underestimation in no-background point source measurements. Finally, for both simulation and real NX systems, the measured PSF parameters were stored in a direction-specific LUT for subsequent spatially-variant PSF modeling during reconstruction.

**Figure 1.**
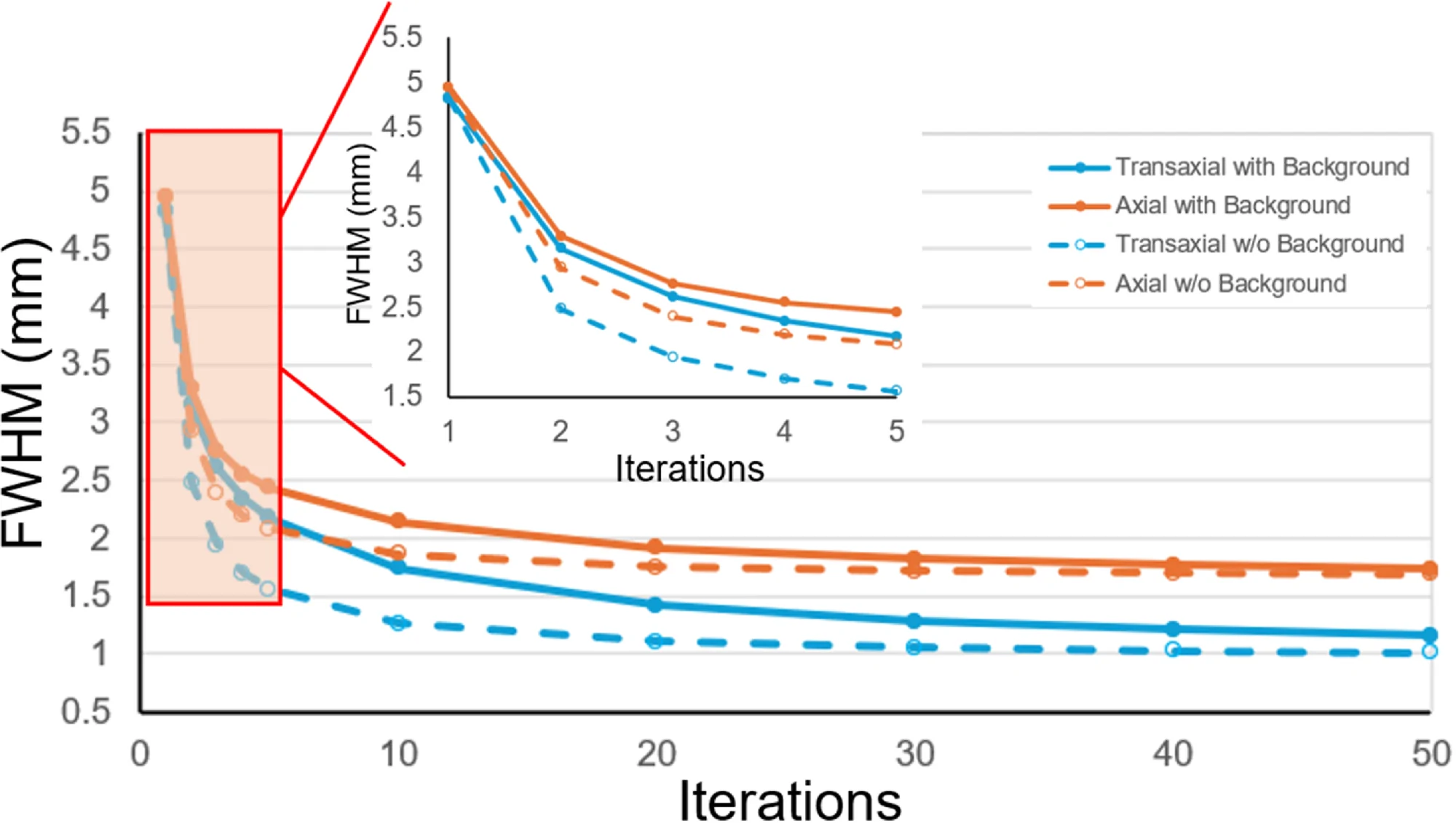
PSF estimation with MLEM iterations from a pure point source at center of FOV and same point source with warm background for 8-layer DOI NX system. Iterations 1–5 were zoomed in to show differences in convergence speed. DOI, depth of interaction; FOV, field of view; MLEM, maximum-likelihood expectation-maximization; NX, NeuroEXPLORER; PSF, point spread function.

A spatially-variant 3D Gaussian convolution operator was implemented in YRT-PET using the measured PSF LUT. During initialization, the LUT is loaded and local FWHM values are converted to voxel-domain Gaussian kernels for both forward and transposed operators. For each voxel, nearest-neighbor interpolation in the LUT is used to select the local kernel parameters before applying convolution.

The forward operator is defined as:

\begin{align*} g\left(i,j,k\right) =&amp; \hspace{-3pt}\sum_{\scriptstyle \Delta x = -k_x}^{\scriptstyle k_x}\!\sum_{\scriptstyle \Delta y = -k_y}^{\scriptstyle k_y}\!\sum_{\scriptstyle \Delta z = -k_z}^{\scriptstyle k_z} \hspace{-2pt}f\left(i+\Delta x, j+\Delta y, k+\Delta z\right)\nonumber\\ &amp;\times h\!\left(i,j,k;\Delta x,\Delta y,\Delta z\right),\end{align*}

where f is the input image, g is the output image after PSF modeling, and $h(i,j,k;\Delta x,\Delta y,\Delta z)$ is the spatially-variant kernel centered at voxel (i,j,k) and evaluated at offset $(\Delta x,\Delta y,\Delta z)$. The kernel half-sizes $k_x$, $k_y$, and $k_z$ define the finite support in each direction. In this study, we define the coordinate system such that the Z-axis corresponds to the scanner axial direction, the X-axis is oriented horizontally with respect to the ground, and the Y-axis is oriented vertically with respect to the ground.

The transposed operator used in backprojection is written as

\begin{align*} g\left(p,q,r\right) = &amp;\hspace{-4pt}\sum_{\scriptstyle \Delta x = -k_x}^{\scriptstyle k_x}\!\sum_{\scriptstyle \Delta y = -k_y}^{\scriptstyle k_y}\!\sum_{\scriptstyle \Delta z = -k_z}^{\scriptstyle k_z} \hspace{-3pt}f\left(p-\Delta x, q-\Delta y, r-\Delta z\right)\nonumber\\ &amp;\times h\!\left(p-\Delta x, q-\Delta y, r-\Delta z;\Delta x,\Delta y,\Delta z\right).\end{align*}

This adjoint form ensures consistency between forward and backward operations within iterative reconstruction. To improve runtime performance, both operators were implemented with GPU parallelization, and circular boundary conditions were used for all convolutions.

## 3. Evaluation

To evaluate the impact of DOI and spatially-variant PSF modeling on NX, we used both MCS and physical phantom datasets and one real NHP scan. All reconstructions were performed using OSEM (Hudson and Larkin 1994) with 10 subsets, including time-of-flight, attenuation, and normalization corrections. The voxel size was 0.6 mm in the transaxial direction and 0.8 mm in the axial direction.

Two PSF modeling strategies were compared against a baseline no-PSF reconstruction (noPSF): the implemented spatially-variant PSF model (VarPSF) and a spatially-invariant PSF model (SinglePSF) that always uses the PSF measured at the center of the FOV. An anti-aliasing filter implemented as a separable 3D Gaussian convolution with FWHM values of 0.90 mm in-plane and 1.66 mm through-plane was included in the forward model for all noPSF results to mitigate discretization artifacts.

### 3.1. Simulated and physical Derenzo phantoms

A simulated mini-Derenzo phantom dataset was generated using uMIP MCS with true coincidences only (no randoms, no attenuation, no scatter, and no background). The total phantom activity was set to 18.5 MBq, and each scan duration was 2 min. The simulated acquisition was selected to provide adequate counts for assessing rod resolvability while limiting the effects of noise. The phantom included hot rods with diameters of 1.2, 1.6, 2.4, 3.2, 4.0, and 4.8 mm, with an active rod length of 40 mm for each sector. Three acquisitions were simulated to form the evaluation dataset: (i) phantom placed at the center of the scanner FOV, with rods parallel to the axial direction (Z); (ii) phantom shifted by 150 mm along the X direction; and (iii) phantom shifted by 150 mm along the Z direction after rotating the phantom by 90$ ^{\circ}$ so that rods were parallel to the horizontal X direction. An additional intermediate DOI configuration (4DOI) was evaluated specifically for the 150 mm X-offset condition (ii), where DOI-related parallax effects are more pronounced, to assess the incremental performance between the 2DOI and 8DOI configurations. Using the same reduced-DOI reassignment framework described in section 2.2, the simulated 8DOI data were mapped to four uniform DOI layers, and reconstructed using the corresponding scanner definition with normalization factors recalculated for the 4DOI configuration.

A physical mini-Derenzo phantom with the same geometry as the simulated Derenzo phantom was scanned on the real NX scanner. Two phantom positions were acquired at the center of FOV and at 150 mm offset along Y direction to match the simulated configurations. The phantom was filled with ^18^F-FDG with a total activity approximately 71 MBq and scanned for 6 min for each acquisition. A CT scan acquired before PET was used for attenuation correction. Physics corrections were applied during reconstruction, except for scatter and random corrections.

For quantitative analysis, the peak-to-valley ratio (PVR) was computed after summing 10 central slices using an open-source semi-automatic hot-spot resolvability tool (Toussaint *et al* 2024), which extracts valleys and peaks from multi-direction line profiles to improve robustness to noise and reports the sector-wise mean PVR. Peak and valley intensities were estimated by averaging profile intensities over the central 50% of each geometrically defined peak and valley region. Then, PVR was calculated as the average of the peak mean intensity divided by the valley mean intensity. This procedure was applied consistently to all simulated and physical Derenzo datasets. For comparison, the 2.4 mm rod region is used, as it is the smallest rod size that can be resolved with VarPSF across all DOI settings and phantom locations. In addition, for the simulated mini-Derenzo dataset, the normalized root mean square error (NRMSE) was computed with respect to the corresponding ground-truth activity image. The RMSE was calculated voxel-wise within the cropped evaluation volume and normalized by the intensity range of the ground-truth, defined as the difference between the maximum and minimum ground-truth voxel values. Because the simulated datasets contained only true coincidences, NRMSE primarily reflects differences in spatial resolution and structural recovery, while also partially reflecting noise properties. Both metrics were analyzed across iterations for different PSF and DOI configurations.

### 3.2. Simulated high resolution brain phantom

A simulated dataset generated from a real 3D-printed high-resolution brain phantom (Lo *et al* 2026) was used to evaluate fine cortical structures in the occipital lobe. The phantom was positioned 100 mm off-center along the Y direction, representing a typical brain position/coverage in NX imaging. The total activity was 0.5 MBq with an acquisition duration of 5 min, and activity was assigned only to gray-matter regions.

Image resolvability was quantified using the resolution-difference metric Ψ, described in Zhang *et al* (2026), which is calculated by non-linear least-squares Gaussian fitting between the high-resolution mask and reconstructed image:

\begin{align*} \Psi = \arg\min_{\psi} \left\lVert M * \psi - R \right\rVert_2^2,\end{align*}

where M is the high-resolution ground-truth activity distribution used to generate the simulated brain phantom, R is the reconstructed image, and ψ is a Gaussian blur kernel parameterized by transverse and axial FWHM components. Here, Ψ denotes the fitted Gaussian kernel that minimizes the sum of squared differences between the blurred mask and the reconstructed image. The transverse and axial FWHM values of Ψ were used as estimates of the resolution difference between the high-resolution mask and the reconstructed image. Smaller FWHM values indicate less blur and therefore better effective spatial resolution. To emphasize the effect of spatially-variant PSF under off-center conditions, the primary reported Ψ result in this work is the transverse FWHM component ($\Psi_{\mathrm{trans}}$), which is most sensitive to resolution changes for the Y-offset phantom. Ψ was analyzed across iterations 1 to 24 for all different PSF and DOI configurations.

In addition to the Ψ analysis, we performed a qualitative comparison between reconstructed images for each PSF/DOI method. The number of iterations was selected for each image by maximizing the ratio between the mean and standard deviation within a brain mask (mean-to-SD ratio) extracted from the ground-truth phantom after one-voxel erosion. This ratio was used solely as a practical criterion for selecting images for visual comparison, rather than as an independent noise metric or a means of matching noise levels across configurations.

### 3.3. *In-vivo* NHP ^11^C-UCB-J scan

We evaluated reconstruction methods *in vivo* on a NHP study performed on the NX scanner and using the ^11^C-UCB-J tracer for synaptic density imaging (Nabulsi *et al* 2016). Reconstructions were performed on static PET data acquired from 30 to 45 min post-injection. The injected dose was 181 MBq, and the brain was positioned 70 mm transaxially and 170 mm axially from the center of the FOV. A CT scan acquired before PET was used for attenuation correction, and a separate T1-weighted MRI scan was performed for region-of-interest (ROI) analysis. Physics corrections were applied during reconstruction, except for scatter and random corrections.

For quantitative evaluation, a standardized NHP brain ROI template was registered to the MRI and then aligned with the PET coordinate system (Seidlitz *et al* 2018). The ROIs used for quantitative analysis were the nucleus accumbens (NAc) and caudate, which are high-uptake subcortical regions with relatively compact anatomy, and cerebral white matter (WM), which provides a lower-uptake reference region and a local estimate of image noise. A two-voxel erosion was applied to the WM ROI to reduce partial-volume effects. Two metrics were calculated for performance comparison between different reconstructions. Contrast was defined as the activity ratio between NAc and WM. The image noise level was quantified using the coefficient of variation (COV), defined as the standard deviation divided by the mean within WM. Metrics were analyzed across OSEM iterations 1 to 12 (10 subsets) for all PSF and DOI configurations.

## 4. Results

In this section, we first study the effect of background in PSF estimation and report the PSF LUTs for different DOI NX systems. Then, we present the simulated and physical Derenzo phantoms, the simulated brain phantom, and the *in-vivo* NHP scan results.

### 4.1. PSF measurements

We compared two PSF measurement strategies for point-source reconstruction: estimation from an MCS point source located in center of 8DOI system without background and estimation from the same point source with warm background (90% activity concentration). As shown in figure 1, the background-included estimation decreases more gradually across iterations and stabilized around 1.2 mm transaxially and 1.8 mm axially. In contrast, the no-background estimation drops rapidly and can underestimate the PSF FWHM, with transverse FWHM reaching about 1.0 mm. Because the early-iteration no-background estimates are closer to the converged background-included values, 10 MLEM iterations were empirically selected for real NX PSF estimation to reduce potential underestimation of the PSF width. The zoomed view of iterations 1–5 highlights this early fast decline in the no-background case.

Figure 2 shows the measured PSF LUTs for different DOI configurations. For characterization of radial spatial variation, point sources positioned along the X axis were used, and the FWHM measured in the X direction was reported as the radial FWHM at each offset. Improved DOI resolution yields smaller PSF FWHM values overall and markedly better radial uniformity across the FOV. In the noDOI system, the radial FWHM increases from 1.5 to 6.8 mm as the offset increases from 0 to 200 mm, whereas in the 2DOI system it increases from 1.4 to 4.3 mm, and in the 8DOI system it increases only from 1.3 to 2.1 mm. In addition, the axial FWHM is larger near the FOV center, which is attributed to DOI effects in large ring-difference lines of response, consistent with the reported NX system (Li *et al* 2024). This observation is in agreement with the axial FWHM uniformity results: improved DOI resolution leads to better axial uniformity, and axial FWHM values at a 200 mm Z-offset are similar across DOI configurations.

**Figure 2.**
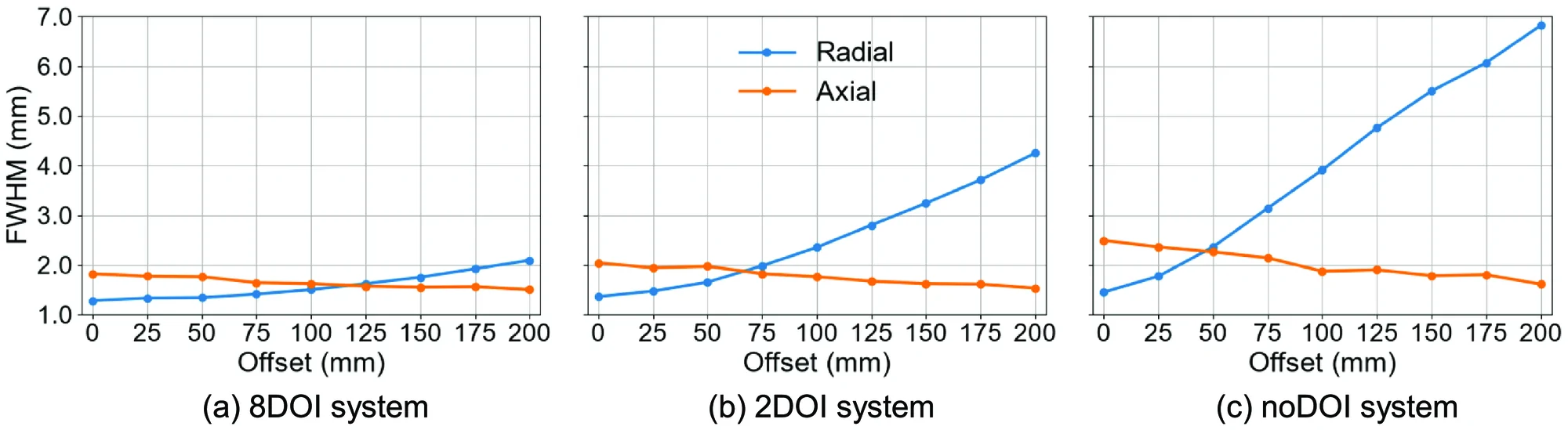
Spatial variation of PSF FWHM measured from the simulated NX system in the radial and axial directions for different DOI configurations. (a) 8-layer DOI NX system, (b) 2-layer DOI NX system, (c) 1-layer, no-DOI NX system. For radial FWHM, the offset denotes the X-direction distance from the FOV center, and the FWHM was measured along the same X direction. For axial FWHM, the offset denotes the axial (Z) distance from the FOV center (with zero radial offset). DOI, depth of interaction; FOV, field of view; FWHM, full width at half maximum; NX, NeuroEXPLORER; PSF, point spread function.

### 4.2. Simulated Derenzo phantom study

#### 4.2.1. Derenzo at center FOV

Figure 3(a) summarizes the convergence behavior of PVR for the center-FOV Derenzo study. Across all DOI configurations, both VarPSF and SinglePSF outperform noPSF in PVR, and VarPSF consistently achieves the best final PVR after convergence. Under realistic NX clinical reconstruction configurations (8DOI, 7 iterations, and 10 subsets), the performance difference between VarPSF and SinglePSF is minimal. In contrast, the benefit of VarPSF becomes most pronounced in the noDOI and 2DOI settings, indicating that spatially-variant PSF modeling can partially compensate for the loss of DOI information. The NRMSE results show trends consistent with the PVR analysis (see supplementary figure S1(a)).

**Figure 3.**
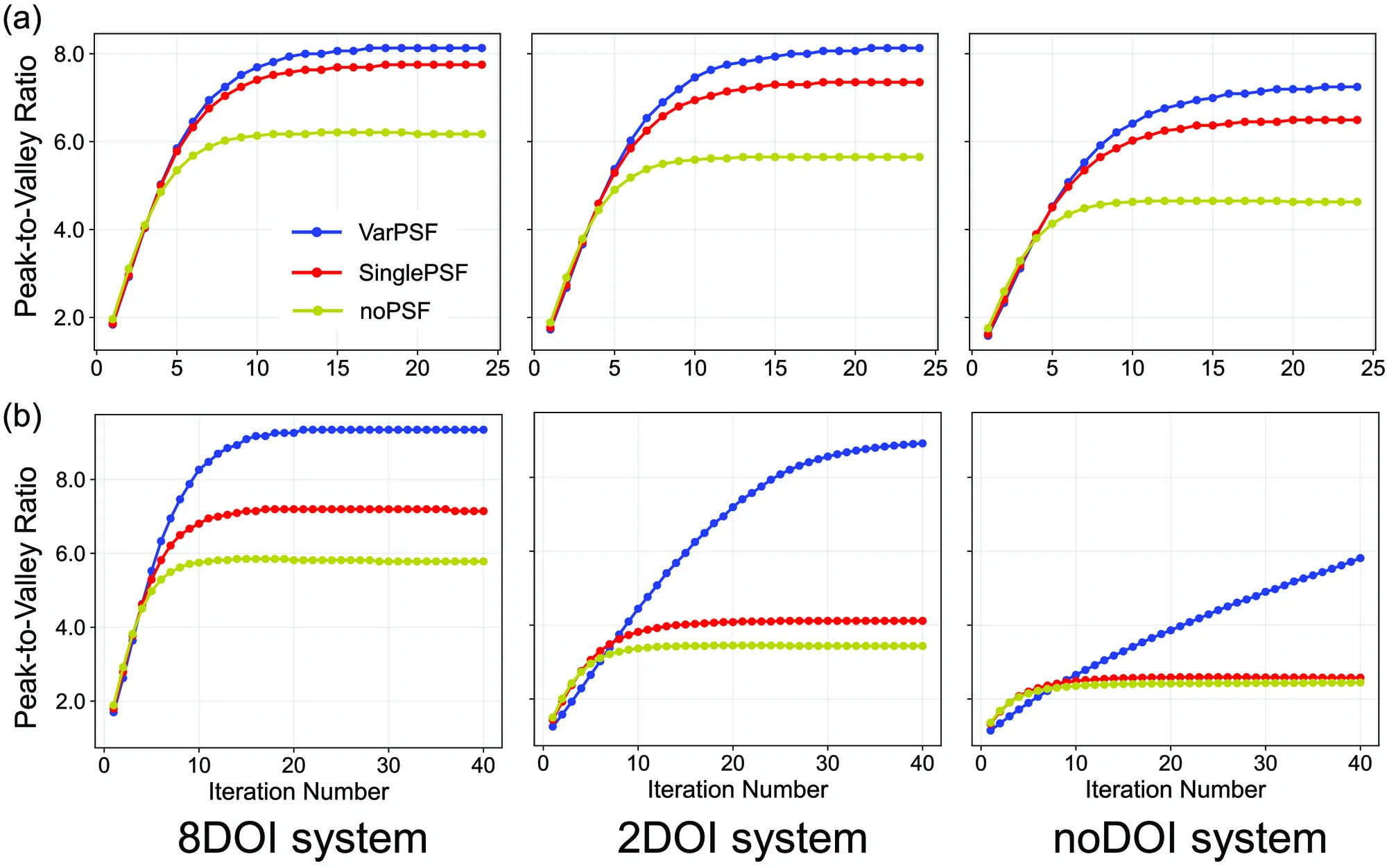
PVR results for simulated Derenzo phantom using different PSF models: (a) at center of FOV and (b) at a 150 mm offset in the X direction. Columns show the 8DOI, 2DOI, and noDOI configurations of the NX system, respectively. A supplementary comparison including the additional 4DOI configuration at the same X offset is provided in Supplementary figure S2. DOI, depth of interaction; FOV, field of view; NX, NeuroEXPLORER; PSF, point spread function; PVR, peak-to-valley ratio.

#### 4.2.2. Derenzo with 150 mm X offset

Figure 3(b) summarizes the PVR convergence behavior for the off-center Derenzo study with a 150 mm X offset. Due to spatial-resolution nonuniformity at large radial offsets, VarPSF achieves higher PVR values than SinglePSF and noPSF at the final evaluated iteration across all DOI configurations. In general, more gain is observed with reduced DOI resolution. Although SinglePSF remains better than noPSF, its benefit is clearly reduced compared with the center-FOV case, where the fixed center-measured PSF is more representative of local system response. In addition, VarPSF shows slower convergence than the other models but achieves higher PVR values within the evaluated iteration range. The NRMSE results show trends consistent with the PVR analysis (see supplementary figure S1(b)). Supplementary figure S2 compares the additional 4DOI configuration with 8DOI, 2DOI, and noDOI at the same offset. Across all PSF models, 4DOI showed intermediate performance between 8DOI and 2DOI, with its PVR at the final evaluated iteration generally closer to that of 8DOI.

#### 4.2.3. Derenzo with 150 mm Z offset

Figure 4 evaluates PSF performance for the noDOI system with a 150 mm Z offset. In addition to SinglePSF which used center FOV PSF estimation, we introduced two variants: SinglePSF- and SinglePSF+. SinglePSF- uses an intentionally smaller spatially-invariant PSF parameter set than the PSF LUT value at the center of FOV (axial PSF FWHM = 1.5 mm, 1.0 mm underestimation), whereas SinglePSF+ uses an intentionally larger PSF parameter set (axial PSF FWHM = 3.5 mm, 1.0 mm overestimation). The goal is to isolate PSF effects and assess how underestimating or overestimating PSF impacts reconstruction performance.

**Figure 4.**
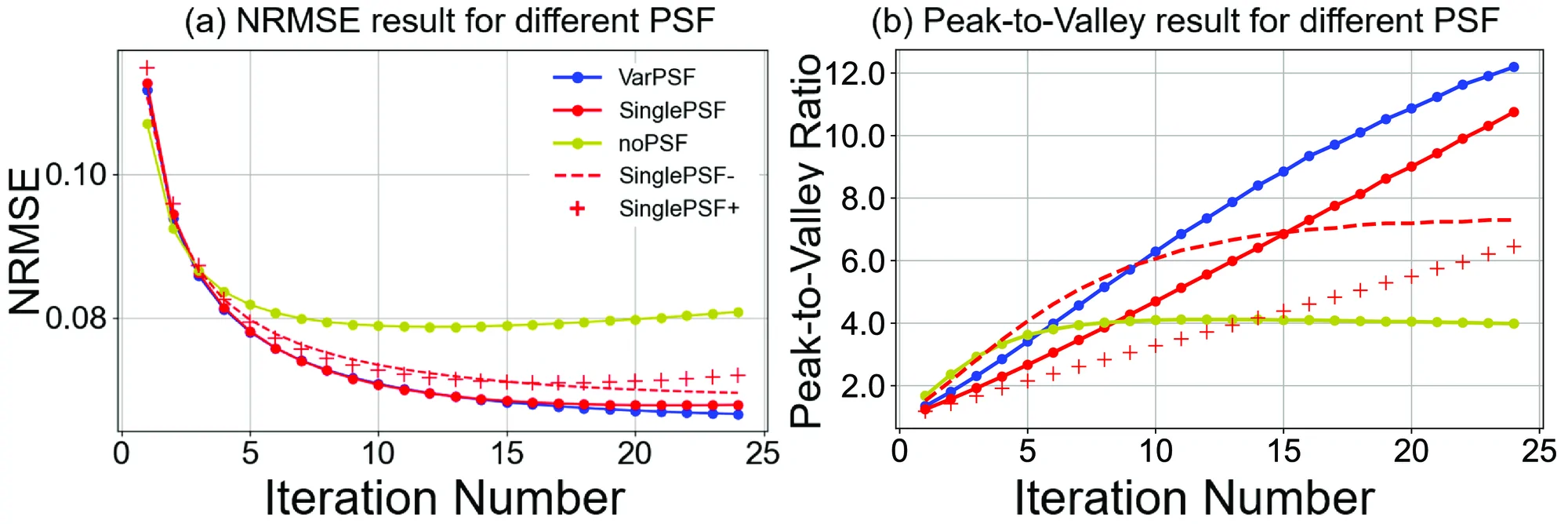
NRMSE (a) and PVR (b) results for simulated Derenzo phantom with a 150 mm offset in the Z direction using different PSF models in the noDOI NX system. SinglePSF- and SinglePSF+ indicate PSF underestimation and overestimation, respectively. DOI, depth of interaction; NRMSE, normalized root mean square error; NX, NeuroEXPLORER; PSF, point spread function; PVR, peak-to-valley ratio.

From the NRMSE curves in figure 4(a), VarPSF outperforms SinglePSF and noPSF. In addition, PSF mismatch degrades performance compared with properly matched VarPSF modeling. In particular, both SinglePSF- and SinglePSF+ show worse final NRMSE than VarPSF and SinglePSF, indicating that either underestimation or overestimation of PSF can reduce quantitative accuracy.

The PVR curves in figure 4(b) show a consistent trend. VarPSF outperforms all other methods, and both SinglePSF- and SinglePSF+ degrade final PVR relative to SinglePSF, confirming that inaccurate PSF parameters reduce contrast recovery. Overall, these results indicate that PSF measurement accuracy is critical, and that both PSF underestimation and overestimation negatively affect image quality.

### 4.3. Physical Derenzo phantom study

#### 4.3.1. Derenzo at center FOV

Figure 5(a) summarizes the PVR results for the physical Derenzo phantom at the center of the FOV. Consistent with the simulation results, VarPSF provides the highest PVR across DOI configurations at later iterations, with its advantage becoming apparent after approximately 10–15 iterations. Improved DOI resolution leads to higher PVR for the same PSF configuration, following the order 8DOI, 2DOI, and noDOI. However, under realistic NX clinical reconstruction settings, the performance difference between VarPSF and SinglePSF remains marginal. Compared with the simulation results, the physical phantoms show lower overall PVR values and slower convergence, potentially reflecting uncorrected scatter and random contributions, as well as residual differences in DOI response and inter-crystal scatter (ICS) modeling between the simulated and physical systems. These factors may affect PVR and its evolution with iterations, although their individual contributions were not isolated in this study.

**Figure 5.**
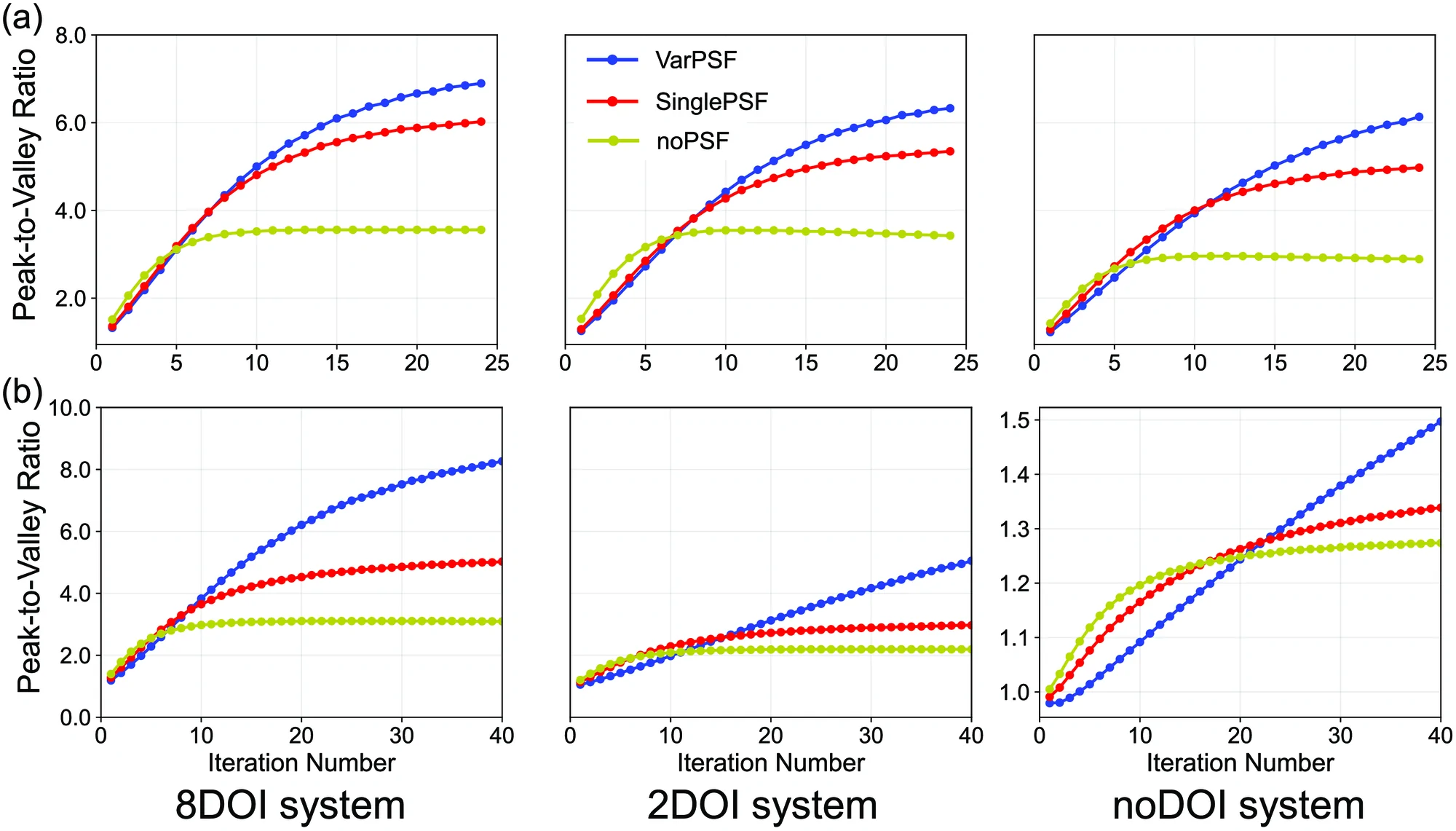
PVR results for the physical Derenzo phantom using different PSF models: (a) at the center of the FOV and (b) at a 150 mm offset in the Y direction. Columns show the 8DOI, 2DOI, and noDOI configurations of the NX system, respectively. Within each row, the plots share the same Y-axis scale, except for the noDOI plot in (b), which uses a separate, narrower range to show differences among PSF configurations more clearly. DOI, depth of interaction; FOV, field of view; NX, NeuroEXPLORER; PSF, point spread function; PVR, peak-to-valley ratio.

#### 4.3.2. Derenzo with 150 mm Y offset

Because the real NX data showed slower convergence than the simulated data, results up to 40 iterations with 10 subsets were analyzed. Figure 5(b) summarizes the PVR results for the physical off-center Derenzo phantom with a 150 mm Y offset. Consistent with the simulated Derenzo results, but requiring more iterations, VarPSF outperforms both SinglePSF and noPSF across all DOI configurations and achieves the highest final PVR. Some VarPSF PVR curves continued to increase at the final iteration; therefore, these results represent performance within the evaluated iteration range rather than fully converged PVR values. At 40 iterations, VarPSF with 2DOI achieves a PVR comparable to SinglePSF with 8DOI, suggesting that the implemented VarPSF model can partially compensate for reduced DOI information in the real system. In the noDOI setting, both noPSF and SinglePSF remain below the Rayleigh-based reference threshold (PVR = 1.36), whereas VarPSF reaches a PVR of 1.50 at 40 iterations. This result indicates that VarPSF improves the resolvability of the 2.4 mm rod sector under the noDOI configuration. Representative VarPSF and SinglePSF reconstructions at 40 iterations are shown in supplementary figure S3. With VarPSF, the 2.4 mm rods are resolved in all three DOI configurations, with rod separation and shape preservation best in 8DOI. Compared with SinglePSF, VarPSF provides clearer rod separation and reduces visible distortion in the 2.4 mm rod area, particularly under reduced DOI encoding, consistent with the PVR results. However, the 1.6 mm rods appear less distinctly resolved with VarPSF than with SinglePSF in 2DOI and noDOI at 40 iterations, potentially reflecting slower recovery of fine structures and sensitivity to residual PSF modeling mismatch.

### 4.4. Simulated brain phantom study

Figure 6 shows the evolution of transverse Ψ-FWHM across iterations for different PSF models and DOI configurations. The Ψ-FWHM decreases with increasing iteration number and gradually reaches convergence, indicating progressive recovery of spatial resolution during iterative reconstruction.

**Figure 6.**
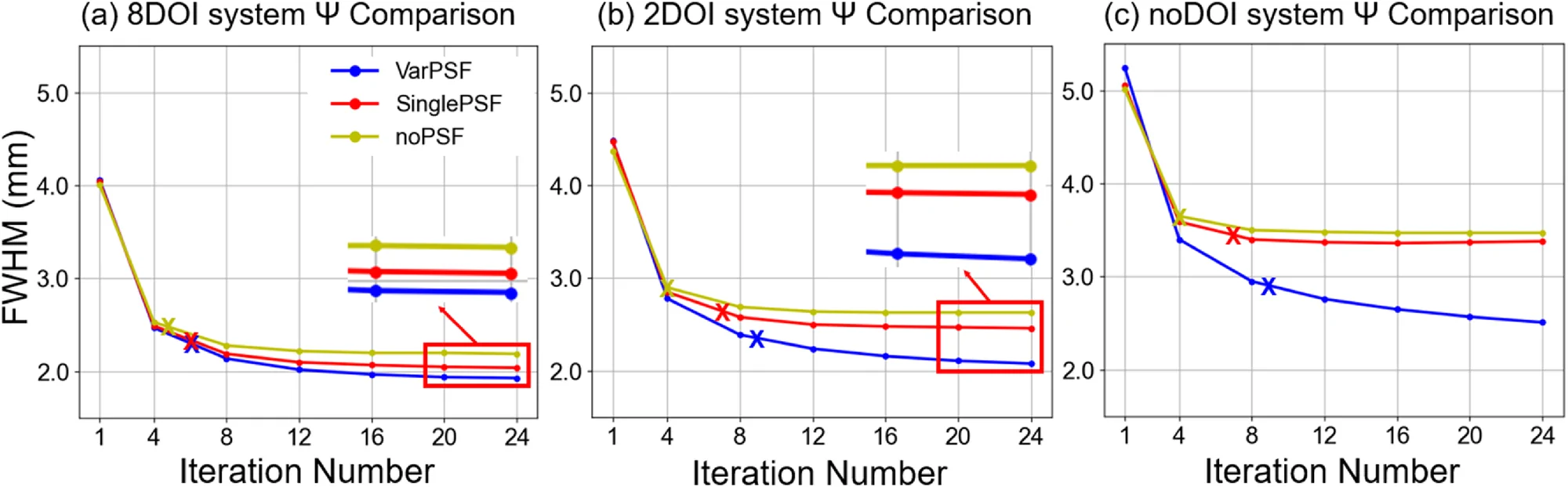
Estimated transverse resolution differences (Ψ) for different PSF models across DOI configurations. (a) 8-layer DOI NX system, (b) 2-layer DOI NX system, (c) 1-layer, no-DOI NX system. The X symbols indicate the iteration where mean-to-SD ratio reaches its maximum for each method. DOI, depth of interaction; NX, NeuroEXPLORER; PSF, point spread function; SD, standard deviation.

Across all DOI configurations, VarPSF consistently outperforms both SinglePSF and noPSF throughout the convergence process and at convergence. Consistent with the Derenzo findings, the relative benefit of VarPSF becomes larger as DOI information becomes less accurate. At clinically relevant reconstruction configurations for NX (8DOI, 7 iterations, 10 subsets), the performance difference between VarPSF and SinglePSF is minimal. In contrast, the performance gap between VarPSF and the other models is most pronounced in the noDOI setting and smaller in 2DOI and 8DOI, showing that spatially-variant PSF modeling can partially compensate for the loss of DOI information in brain phantom reconstruction. When the mean-to-standard deviation (SD) ratio reached its maximum for each method, VarPSF reduced (Ψ) by approximately 0.3 mm relative to SinglePSF and 0.5 mm relative to noPSF in the 2DOI configuration, and by 0.6 mm and 0.8 mm, respectively, in the noDOI configuration.

In figure 6, the X symbols mark the data points at the iteration corresponding to the maximum mean-to-SD ratio for each method. These markers show that with VarPSF, 8DOI reaches its maximum mean-to-SD ratio earlier than 2DOI and noDOI. Within each DOI configuration, VarPSF typically requires more iterations to reach its mean-to-SD ratio peak than SinglePSF and noPSF. Visual comparisons and line profiles at the selected iterations in figure 7 show that, in the noDOI configuration, VarPSF provides better cortical delineation than SinglePSF and noPSF. The profile comparison also shows that, in the 2DOI configuration, VarPSF achieves higher contrast than the other methods.

**Figure 7.**
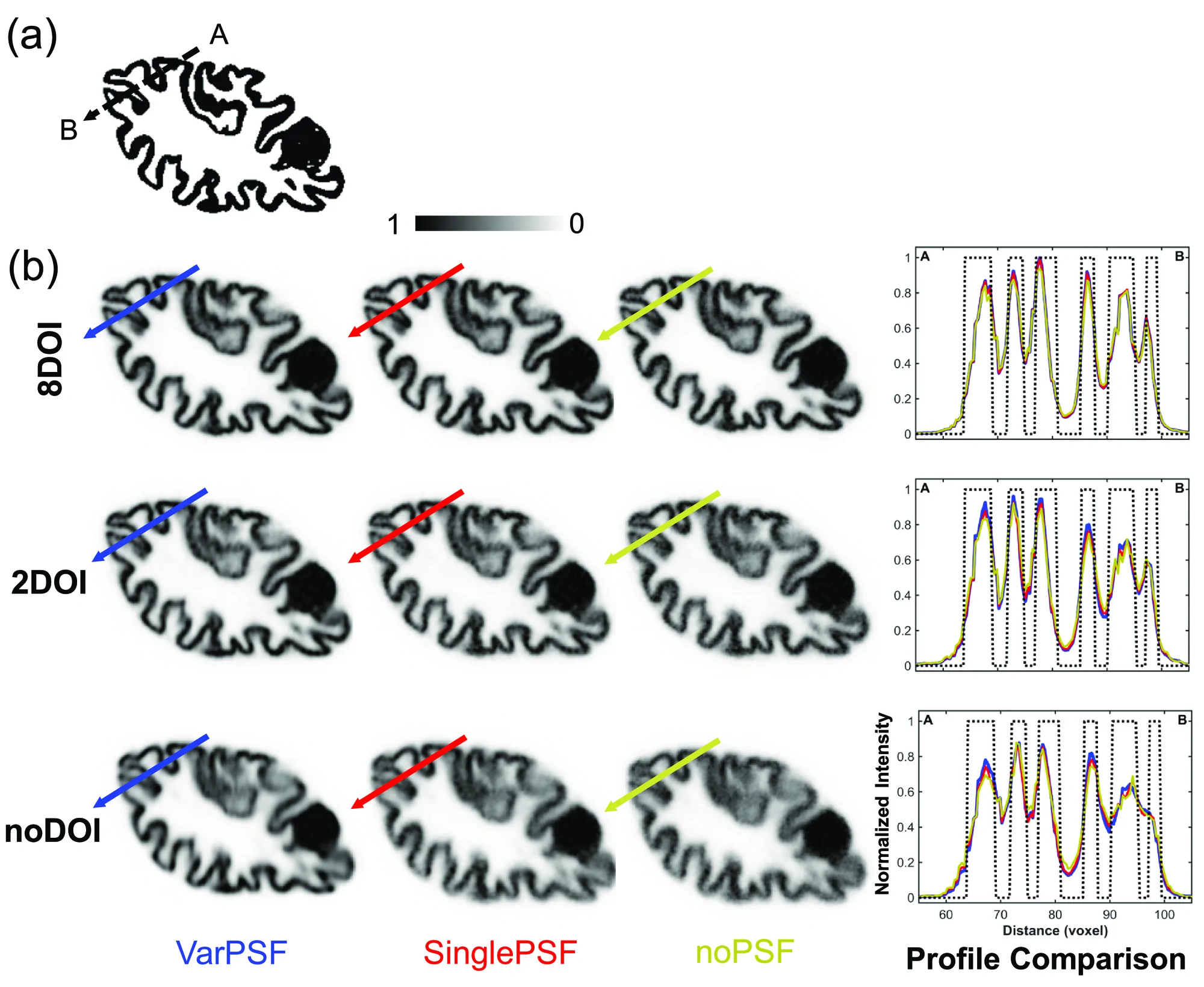
Visual and profile comparisons for the simulated brain phantom across different PSF and DOI configurations of the NX system. Each reconstruction is shown at the iteration that maximizes its mean-to-SD ratio. (a) Ground-truth activity distribution. (b) Reconstructed images and corresponding line profiles for 8DOI, 2DOI, and noDOI (rows), with VarPSF, SinglePSF, and noPSF shown in blue, red, and yellow, respectively. Arrows indicate the profile sampling paths from A to B, corresponding to the left-to-right direction in the profile plots. Black dotted lines show the corresponding ground-truth profiles. DOI, depth of interaction; NX, NeuroEXPLORER; PSF, point spread function; SD, standard deviation.

### 4.5. *In-vivo* NHP study

Figure 8 shows contrast-versus-noise curves calculated from NAc and WM under different PSF and DOI configurations. Under clinically relevant reconstruction configurations (7 iterations, 10 subsets), contrast in the noPSF condition increases with DOI accuracy: 8DOI = 2.27, 2DOI = 2.19, and noDOI = 2.14. At 12 iterations, the corresponding contrast values are 2.28, 2.20, and 2.14, respectively. For all DOI systems, VarPSF achieves higher contrast than both SinglePSF and noPSF at 12 iterations. Consistent with the simulation results, the contrast gain is greater when DOI resolution is less accurate. The final contrast values for VarPSF across DOI systems are close (difference <0.1); this consistency indicates that the implemented VarPSF method provides robust compensation for DOI resolution limitations in real data. VarPSF also achieves a lower COV at 12 iterations than noPSF while maintaining a WM noise level comparable to SinglePSF (within 2%). Supplementary figure S4 presents contrast-noise curves computed using the caudate as an alternative high-uptake region, yielding results consistent with those observed in NAc.

**Figure 8.**
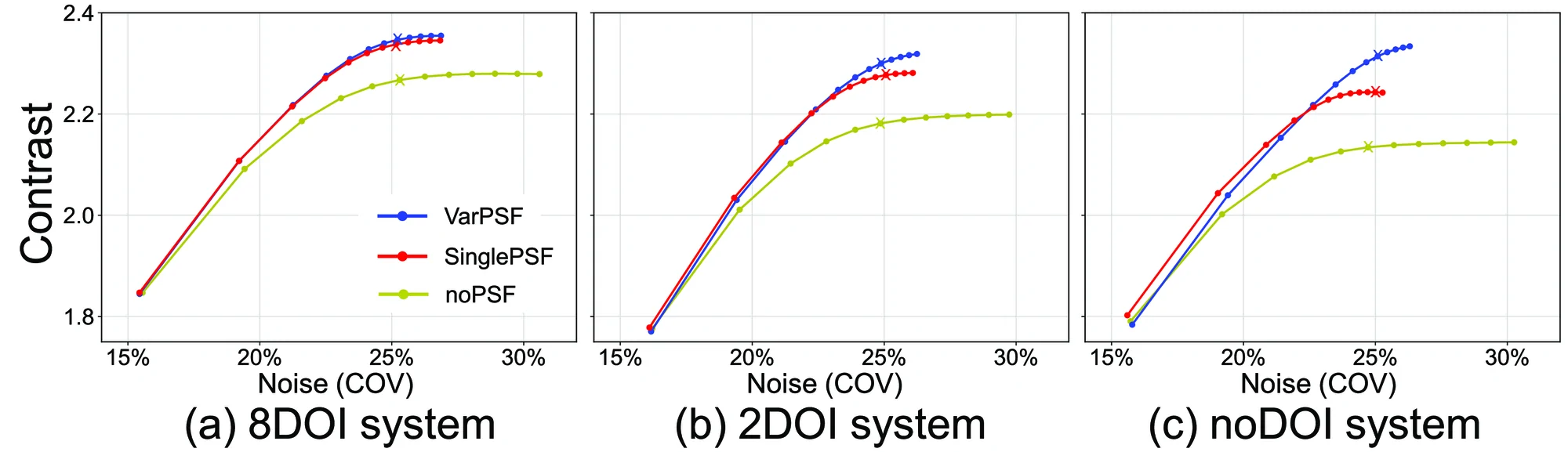
Contrast-versus-noise curves for different PSF models and DOI configurations in the NX system across iteration 1 to 12. Contrast was defined as the activity ratio between NAc and WM, while noise was quantified using the COV within WM. The X symbols denote the iteration at which the noise level is around 25% for each method. COV, coefficient of variation; DOI, depth of interaction; NAc, nucleus accumbens; NX, NeuroEXPLORER; PSF, point spread function; WM, white matter.

For fair visual comparison, we compared images at matched noise level (25%) by selecting, for each method, the iteration indicated by the X markers in figure 8. Visual comparison in figure 9 shows that, under equal-noise condition, VarPSF consistently outperforms SinglePSF and noPSF across 2DOI and noDOI configurations, providing improved spatial resolution, enhanced gray-to-white-matter contrast and increased image sharpness. These improvements enable more precise delineation of subcortical regions with high ^11^C-UCB-J binding (e.g. caudate, inferior colliculi, putamen), and improved definition of the cortical ribbon (as illustrated for anterior cingulate cortex and inferior frontal). Similar to the simulation study, the incremental gain of VarPSF over SinglePSF decreases from noDOI to 2DOI and 8DOI; under the clinically relevant 8DOI configuration, the visual difference between VarPSF and SinglePSF remains limited, indicating that accurate DOI encoding already recovers much of the resolution and contrast loss.

**Figure 9.**
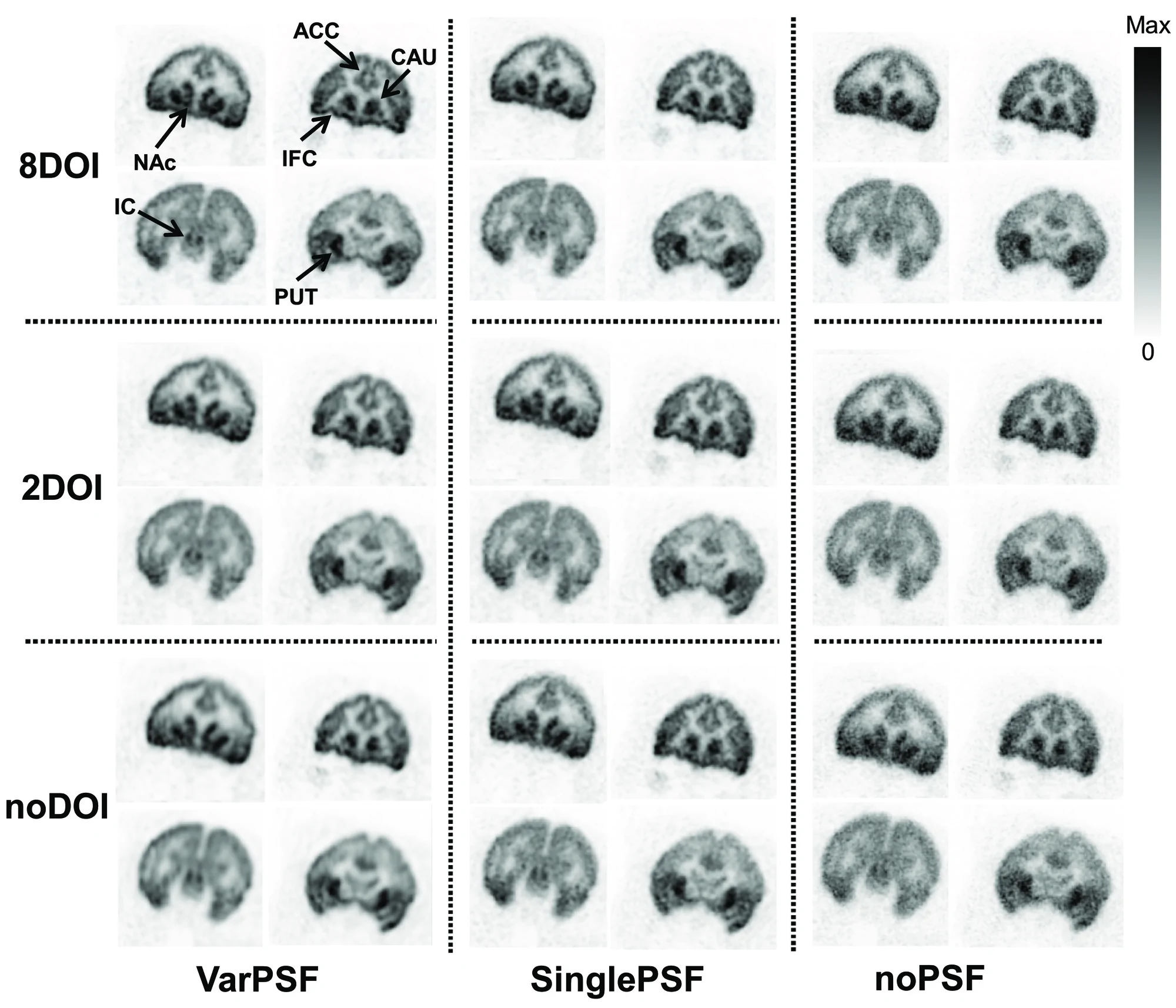
Visual comparison of reconstruction across different PSF and DOI NX systems at a matched noise level, in NHP NX data (^11^C-UCB-J PET 30–45 min summed image). Four axial slices are shown for each DOI setting (8DOI, 2DOI, and noDOI) and PSF configuration (VarPSF, SinglePSF, and noPSF). Arrows highlight brain structures of interest: anterior cingulate cortex (ACC), caudate (CAU), inferior colliculi (IC) in the brainstem, inferior frontal cortex (IFC), nucleus accumbens (NAc), putamen (PUT). DOI, depth of interaction; NHP, non-human primate; NX, NeuroEXPLORER; PET, positron emission tomography; PSF, point spread function.

## 5. Discussion

In this study, we measured the spatially-variant PSF of the NX and investigated the impact of DOI and PSF modeling using both simulation and real NX data. Overall, the results confirm the important role of DOI in high-resolution dedicated brain PET, particularly for maintaining uniform resolution across the FOV. Furthermore, improved DOI resolution tended to converge faster in OSEM, which is practically important for iterative reconstruction because noise increases with the number of iterations (Rahmim *et al* 2013). Results also show that the implemented VarPSF model consistently improves reconstruction performance relative to SinglePSF and noPSF, with the largest benefit observed when DOI resolution is limited. Because both DOI encoding and PSF modeling affect the noise-resolution trade-off during iterative reconstruction, we evaluated performance across iteration number and, in the NHP study, at approximately matched noise conditions. More generally, the optimal iteration setting for NX imaging is also expected to depend on additional factors such as count level and tracer kinetics in human studies (Hu *et al* 2026). Accordingly, the principal contribution of this work is the open-source implementation of VarPSF in YRT-PET together with a controlled evaluation of its interaction with DOI resolution in NX, demonstrating that the benefit of spatially-variant resolution modeling increases as DOI resolution is reduced and can partially compensate for limited detector-level DOI encoding.

We also investigated SinglePSF variations with intentionally underestimated (SinglePSF-) and overestimated (SinglePSF+) PSF parameters. In figure 4(b), the PVR curve of SinglePSF+ does not plateau within 24 iterations, suggesting that an overly large PSF can continue to increase apparent resolution and contrast metrics with additional iterations. However, this should not be interpreted as improved quantitative performance, since an excessively large PSF model can produce over-sharpened images and ringing artifacts, which may degrade image fidelity and quantitative accuracy (Tong *et al* 2010). Nevertheless, a broader SinglePSF kernel than the one measured in the center of the scanner can be used to match the average brain position in typical scans.

One strength of this work lies in the implementation of the spatially-variant PSF model within YRT-PET, an open-source PET reconstruction platform (Najmaoui *et al* 2026). Because the model is parameterized using PSF measurements obtained from point sources, it can in principle be extended to other PET systems. The open-source nature of YRT-PET also promotes broader adoption, reproducibility, and adaptation of spatially-variant PSF modeling across PET platforms.

The effects of DOI and PSF modeling on image quality were evaluated using both simulations and real NX acquisitions. The simulation studies used the GPU-based uMIP MCS platform, which allowed the NX system response to be measured from point sources with warm background, a measurement that is difficult to perform experimentally. To assess agreement between simulation and the physical scanner, point-source FWHM was also measured according to the National Electrical Manufacturers Association NU 2–2018 standard using filtered backprojection. As shown in Supplementary figure S5, the simulated and real NX systems show consistent radial and axial FWHM trends, supporting good agreement between the uMIP model and the real scanner. However, the real system shows slightly larger FWHM values, potentially reflecting detector effects, such as ICS (Zhang *et al* 2026), that are not fully captured in simulation. Although the MCS incorporates DOI uncertainty based on measured system-average performance, this approximation may not fully reproduce the actual DOI response across detector elements and interaction depths. The simulation study also clarified the effect of background on PSF measurement: pure point-source measurements tended to underestimate the PSF with increasing iteration number, whereas point sources with warm background provided more stable estimates. This insight motivated the ad hoc early-stop strategy used in real-system PSF estimation, so that the no-background measurements better approximate the PSF values expected under more realistic conditions.

Our findings highlighted the practical implications of the VarPSF implementation used in this study. Based on the PSF measurements in figure 2, spatial resolution variation within the brain region is relatively small for the 8DOI configuration. As a result, it is often possible to identify a well-tuned single PSF model that provides near-optimal performance for standard brain reconstructions. However, a key advantage of the NX system is its extended axial FOV, which enables simultaneous brain and neck imaging applications, such as carotid artery image-derived input function estimation (Volpi *et al* 2025). In these scenarios, maintaining consistent image quality across a larger spatial extent can become beneficial. The implemented VarPSF approach offers potential advantages over the SinglePSF model, by providing spatially adaptive resolution modeling, which may improve quantitative accuracy and robustness when both brain and neck regions are involved.

Our findings also provide guidance for future brain PET system design. Although VarPSF substantially improved image quality in the noDOI configuration, it did not recover the image quality achieved with 8DOI, indicating that spatially-variant PSF modeling can partially compensate for degraded DOI information, but cannot fully replace detector-level DOI encoding. Beyond the limitations of the Gaussian approximation, image-domain PSF modeling does not capture the LOR-dependent resolution degradation and therefore cannot be expected to fully capture all components of resolution degradation. Hybrid projection/image-domain PSF modeling could represent these effects more accurately and potentially improve resolution recovery, although at additional computational cost (Deller *et al* 2021). Even with more complete resolution modeling, the distribution of unmeasured interaction depths would be modeled statistically rather than providing the event-specific information provided by DOI measurements. We therefore do not expect improved PSF modeling alone to fully replace DOI encoding with finite counts and practical reconstruction settings. In addition to these modeling considerations, within the same DOI configuration, VarPSF generally showed slower convergence than SinglePSF and noPSF, highlighting the additional iterations required for resolution recovery. Nevertheless, 2DOI combined with VarPSF achieved performance closer to that of 8DOI, suggesting a potential compromise between DOI capability and imaging performance. In the simulated Derenzo study at a 150 mm X offset (Supplementary figure S2), 4DOI performance was intermediate between 2DOI and 8DOI and generally closer to 8DOI, suggesting diminishing gains from finer DOI encoding under this condition. This may be particularly relevant for emerging ultra-high-resolution brain PET designs such as the no-DOI system reported in Doyon *et al* (2025), which achieves excellent central resolution. Our results motivate evaluating whether modest DOI capability, such as 2-layer DOI, could further improve off-center performance and resolution uniformity in such systems. These potential improvements must be weighed against the hardware requirements of DOI implementation. For example, dual-ended readout can provide precise DOI localization but requires additional photodetectors and associated electronics, increasing hardware cost and complexity (Liu *et al* 2022). Taken together, our findings suggest that 2DOI combined with VarPSF is a promising starting point for future detector-design studies aimed at balancing image quality and hardware complexity, although further evaluation is needed to determine the optimal DOI configuration.

A limitation of the current study is that the implemented spatially-variant PSF was modeled using a symmetric 3D Gaussian fitting together with nearest-neighbor interpolation to cover the full FOV. Although this approach was sufficient to demonstrate the benefit of system-specific spatially-variant resolution modeling, it may not fully capture the true local system response and could introduce modeling bias, especially in regions where the PSF shape is more complex (Gravel *et al* 2019). The representative profiles in Supplementary figure S6 show generally close agreement in shape between the reconstructed point-source profiles and the fitted Gaussian model, with the largest discrepancy observed for noDOI at the 200 mm X offset. The visibly asymmetric profile in this case illustrates the limitation of the symmetric Gaussian approximation at large radial offsets without DOI information. Future work will therefore investigate more flexible PSF representations, such as Gaussian mixture models (Zeng *et al* 2021), as well as different interpolation methods and the spatial distribution and spacing of point-source measurements to construct PSF LUTs that more accurately capture spatial variations in the system response. In addition, previous work has shown that ICS events can alter the effective system resolution (Zhang *et al* 2026); because NX has the ability to identify these events, incorporating ICS-dependent resolution effects into the spatially-variant PSF model is another important direction for improving resolution modeling while preserving system sensitivity.

A separate limitation concerns the interpretation of the simulated DOI configurations. Although the MCS included NX detector-response modeling and DOI uncertainty, the 4DOI, 2DOI and noDOI configurations were generated by remapping DOI information from the original NX 8DOI data rather than by simulating physically redesigned detector hardware. Thus, these results isolate the effect of DOI information granularity within the NX detector geometry and should not be interpreted as absolute performance predictions for newly constructed 4DOI, 2DOI or noDOI systems, whose detector geometry and readout characteristics may differ. Finally, the quantitative interpretation of the physical phantom and NHP results is limited by the omission of scatter and random corrections. Although these corrections were omitted consistently across all DOI and PSF configurations, their absence may bias ROI metrics and affect convergence behavior. The findings therefore reflect relative performance under these uncorrected conditions, and their applicability to fully corrected reconstructions requires further evaluation. Future work will incorporate NX-specific scatter and random estimation into YRT-PET to support quantitative reconstruction.

## 6. Conclusion

In this study, we implemented a spatially-variant PSF model for NX in the open-source YRT-PET platform and systematically evaluated the interplay between DOI encoding and resolution modeling using simulated and physical datasets. VarPSF improved resolution recovery relative to SinglePSF and noPSF, with larger benefits under reduced DOI encoding and at large radial offsets. Its incremental benefit over SinglePSF was limited under the evaluated 8DOI brain imaging conditions at clinically relevant iteration settings. Combining 2DOI with VarPSF narrowed the performance gap relative to 8DOI, while the additional off-center simulated Derenzo study showed 4DOI performance between that of 2DOI and 8DOI. These findings highlight the complementary roles of DOI encoding and spatially-variant resolution modeling and suggest that combining modest DOI capability with accurate PSF modeling may offer a practical balance between detector complexity and reconstructed image quality. Overall, this work demonstrates the importance of modeling the spatially-variant PSF for high-performance brain PET imaging, especially for PET systems with limited DOI resolution. Further validation is needed to establish this balance in future detector designs and with scatter- and randoms-corrected reconstructions.

## Data Availability

The data cannot be made publicly available upon publication because they are owned by a third party and the terms of use prevent public distribution. The data that support the findings of this study are available upon reasonable request from the authors. Supplementary figures available at https://doi.org/10.1088/1361-6560/aeafa5/data1.
